# Developing a core outcome set for acetabular fractures: a systematic review (part I)

**DOI:** 10.1186/s13643-025-02824-0

**Published:** 2025-04-09

**Authors:** Denise Schulz, Catharina Gaeth, Martin C. Jordan, Steven C. Herath, Christopher Spering, Dan Bieler, Joachim Windolf, Anne Neubert

**Affiliations:** 1 Department of Orthopaedics and Traumatology, Medical Faculty and University Hospital Düsseldorf, Heinrich-Heine-University Düsseldorf, Düsseldorf, Germany; 2 TraumaEvidence @ German Society of Trauma Surgery, Berlin, Germany; 3 Department for Trauma Surgery and Orthopaedics, Reconstructive and Hand Surgery, Burn Medicine, German Armed Forces Central Hospital, Koblenz, Germany; 4 Centre of Orthopaedics, Trauma Surgery and Rehabilitative Medicine, University Medicine Greifswald, Greifswald, Germany; 5 Department of Trauma and Reconstructive Surgery, Eberhard Karls University Tuebingen, BG Trauma Center Tuebingen, Tubingen, Germany; 6 Department for Trauma Surgery, Orthopaedics and Plastic Surgery, University Medical Center Göttingen, Göttingen, Germany

**Keywords:** Core outcome set, Outcome reporting, Acetabular fractures, Systematic review

## Abstract

**Background:**

There are indications that clinical studies investigating the surgical treatment of acetabular fractures assess different outcomes. This heterogeneity reduces the comparability of study results and, thus, limits the knowledge generated from research. Core outcome sets (COS) contain a minimum set of outcomes that should be measured in studies investigating a specific disease or injury. A COS for surgically treated acetabular fractures does not yet exist. Therefore, the aim of this study is to identify the reported outcomes in studies investigating the surgical treatment of acetabular fractures.

**Methods:**

Studies including skeletally mature individuals (≥ 16 years) with isolated acetabular fractures treated surgically were included. Studies with polytrauma patients, pathological fractures, additional pelvic fractures, exclusively non-surgical treatment, or juvenile individuals were excluded. Three databases and two clinical trial registries were searched on 15 November 2022. The identified outcomes were grouped and subsequently categorized according to the Core Outcome Measures in Effectiveness Trials Guidelines.

**Results:**

A total of 193 studies were included, which reported a cumulative total of 2581 outcomes. After grouping, 266 unique outcomes were identified. No outcome was examined in all studies. *Pain*, *ability to walk independently*, *range of motion*, *quality of reduction*, and *heterotopic ossification* were the most reported unique outcomes and assessed in at least 60% of included studies. A total of 105 outcomes were only assessed in one of the included studies. Outcomes of all five core areas and 25 outcome domains of the Core Outcome Measures in Effectiveness Trials taxonomy were examined. Furthermore, outcomes were named and defined differently, measured at different time points, and assessed using a variety of measurement instruments.

**Conclusion:**

Overall, this systematic review shows that a wide range of outcomes are measured in studies examining surgical treatment of acetabular fractures. The results of this systematic review will be used in a subsequent study to develop the COS for surgically treated acetabular fractures by using the Delphi method.

**Systematic review registration:**

PROSPERO: CRD42022357644; COMET: 2123.

**Supplementary Information:**

The online version contains supplementary material available at 10.1186/s13643-025-02824-0.

## Background

Acetabular fractures, although relatively rare, with an incidence of 3 to 40 per 100,000 persons per year, represent a complex injury pattern characterized by a bimodal distribution in the population [1–3]. The younger demographic, predominantly male individuals typically experience these injuries due to high-velocity trauma such as motor vehicle accidents or falls from significant heights. In contrast, older adults often sustain these fractures from low-energy mechanisms such as falls from a standing position, which can lead to fragility fractures associated with diminished bone quality [2, 4]. Notably, data from the German Pelvic Registry indicates that over 50% of acetabular fractures occur in individuals aged 60 years and older, with an increasing incidence due to demographic shifts toward an aging population [2, 5, 6]. In elderly, acetabular fractures are associated with higher complication rates and increased mortality compared to other orthopedic injuries, including hip fractures [7, 8]. Khoshbin et al. (2020) emphasize that the risk of mortality following acetabular fractures exceeds that of hip fractures, underscoring the severity and clinical significance of these injuries in older adults [9].

Clinical research on acetabular fractures is essential to advance the understanding of injury mechanisms, optimize treatment strategies, and improve patient outcomes and quality of life. Although there is a substantial body of research literature reporting outcomes of surgically treated acetabular fractures, there is considerable variability in the results and outcomes reported among these studies [10, 11]. These discrepancies can arise from differences in study design, research objectives, and the specific outcomes assessed, ranging from surgical techniques and complication rates to rehabilitation protocols and functional recovery [12, 13]. This heterogeneity significantly impedes the ability to compare, contrast, and synthesize findings, thereby limiting the development of evidence-based clinical guidelines and the identification of superior treatment modalities [14]. The rarity of acetabular fractures exacerbates this problem; individual studies often have small sample sizes that reduce statistical power and the generalizability of findings [1–3]. To address these challenges, the Core Outcome Measures in Effectiveness Trials (COMET) initiative established methods to develop core outcome sets (COS) that can reduce heterogeneity and inconsistency in outcome assessment and reporting. COS are standardized, research-based sets of key outcomes that should be measured and reported in all clinical trials investigating a specific disease or injury, such as acetabular fractures. The implementation of COS allows more study results to be included in the evidence synthesis, resulting in more valuable findings and comparability of studies, thereby improving scientific findings [15, 16]. Currently, there is no COS for surgically treated acetabular fractures, which represents a significant gap in research standardization.

The aim of this systematic review is to analyze the outcomes reported in studies of surgically treated acetabular fractures in skeletally mature individuals. In a subsequent study, the identified outcomes will be used to develop the COS for surgically treated acetabular fractures. The establishment of a COS for acetabular fractures will reduce heterogeneity in outcome reporting, improve evidence synthesis, and ultimately enhance clinical decision-making and patient care.

## Methods

This systematic review is reported according to Preferred Reporting Items for Systematic Reviews and Meta-Analyses (PRISMA) and the Core Outcome Set-STAndards for Reporting (COS-STAR) guidelines [16, 17]. The completed checklists can be found in Additional file 1 and 2. The systematic review was registered via PROSPERO (registration nr. CRD42022357644) and the entire COS for acetabulum fractures project was registered with the COMET database (https://www.comet-initiative.org/Studies/Details/2123). The methodological procedure of the systematic review is based on the COMET Handbook [12]. A detailed version of the methodology is reported in the published protocol by Schulz et al. [18]. There are no amendments to the protocol.

### Eligibility criteria

All studies investigating the surgical treatment of isolated acetabular fractures in patients aged ≥ 16 years were eligible for inclusion. Studies including patients with polytrauma, pathological fractures, additional pelvic fractures (e.g., pelvic ring fractures), sole non-surgical treatment, or skeletally immature patients (< 16 years) were excluded. Furthermore, studies that did not examine outcomes in relation to the surgical treatment of acetabular fractures were not included (e.g., studies of risk factors or diagnostics). Systematic reviews, case reports, biomechanical, cadaveric, animal studies, and studies involving fewer than ten individuals were excluded.

### Search strategy and selection process

The databases MEDLINE via PubMed, Cochrane Central Register of Controlled Trials (CENTRAL), and Web of Science Core Collection were searched from inception to 15. November 2022. For the identification of ongoing or unpublished studies, ClinicalTrials.gov and World Health Organization International Clinical Trials Registry Platform were searched. Furthermore, the references of relevant systematic reviews were searched manually. Only studies published in English or German were eligible, but there were no restrictions regarding the publication date. The search strategies are provided in Additional file 3.

Two authors screened the titles and abstracts and, subsequently, the full texts of the search hits using the Covidence® software [19]. Disagreements were resolved via discussion or if necessary, by a third reviewer.

### Data extraction

Data were extracted with a pre-developed form that was piloted. The data from the included studies were extracted by two authors independently using the Covidence® software [12, 19]. Disagreements were resolved through discussion. Reported outcomes, their definition, time point(s), and measurement instrument(s) were extracted in verbatim.

Outcomes were defined as results reported in the methods or results separately from study and participants characteristics. In many studies, complications were only summarized as one outcome under the term “complications” without specifying the term in more detail. If reported complications were extracted as individual outcomes, and if a measurement instrument contained more than one item, each individual item was extracted as an outcome [20].

As stated in the protocol, the risk for outcome reporting bias was assessed using the Outcome Reporting Bias In Trials (ORBIT) study classification system [18, 21]. However, the entire study to develop a COS for surgically treated acetabular fractures is still ongoing. The results of the outcome reporting bias assessment and the Delphi study will be presented in a subsequent manuscript.

### Data synthesis

In a first step, outcomes that measure the same concept but have different names or definitions were grouped to identify the number of unique outcomes. Afterwards, unique outcomes were categorized using the taxonomy of the COMET initiative [22]. Outcome grouping and categorization were carried out by two authors with methodological or clinical expertise independently using Microsoft Excel. Conflicts were solved via discussion.

A subgroup analysis that illustrated reported outcomes by year of publication (before and after 2000) was conducted.

## Results

**Fig. 1:**
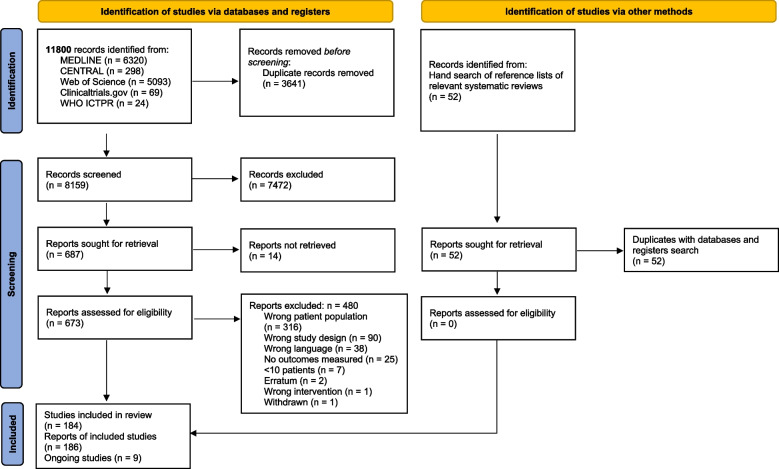
PRISMA flow diagram. Legend: from Page et al. [17]

The search resulted in 11,800 hits. After deduplication, 8159 records were screened by title and abstract, resulting in 673 full texts that were reviewed for inclusion. The manual search did not identify any additional studies for inclusion. Finally, 184 published studies with 186 records [23–208] and 9 ongoing studies [209–217] were included in this systematic review. The search and selection process were documented in a PRISMA flow diagram (Fig. 1) [17].

### Study characteristics

The 184 studies were published in the years 1975 to 2023 and involved a total of 11,321 participants [23–59, 61–82, 84–208]. The 193 included studies are nine randomized controlled trials (RCT) (4.66%), one mixed methods study (RCT & observational study) (0.52%), 66 observational studies with control group (34.20%), and 117 observational studies without control group (60.62%). The studies have been or are being conducted in 36 different countries, most frequently in China (*n* = 35; 18.13%), the USA (*n* = 28; 14.51%), India (*n* = 20; 10.36%), Germany (*n* = 13; 6.74%), and Switzerland (*n* = 9; 4.66%) (Table 1). More detailed study characteristics are provided in Additional file 4.

**Table 1:** Study characteristics

Characteristics	Number of studies (%)
**Study design**	
RCT	9 (4.66)
Mixed methods	1 (0.52)
Observational studies with a control group	66 (34.20)
Observational studies without a control group	117 (60.62)
**Publication year**	
1975–1999	12 (6.22)
2000–2023	172 (89.12)
Ongoing	9 (4.66)
**Country**	
China	35 (18.13)
USA	28 (14.51)
India	20 (10.36)
Germany	13 (6.74)
Switzerland	9 (4.66)
Other	88 (45.60)

*Legend*: *RCT* randomized controlled trial, *USA* United States of America

### Outcomes

Overall, 2581 outcomes were measured cumulatively in 193 studies. The studies measured on average 13.37 outcomes (range 1–36). Grouping of outcomes with diverse names that measured the same concept resulted in a total of 266 unique outcomes. No outcome was assessed in all studies. *Pain* was the most frequently reported outcome (*n* = 158; 81.86%). Other often reported outcomes were *ability to walk independently* (*n* = 140; 72.54%), *range of motion* (*n* = 139; 72.02%), *quality of reduction* (*n* = 127; 65.80%), and *heterotopic ossification* (*n* = 117; 60.62%). In total, 39.47% of the outcomes (*n* = 105) were measured in only one study (e.g., *wounds healing time*, *iatrogenic obturator artery injury*, *screw irritation*, and *erectile dysfunction*).

### Subgroup analysis

Of the 193 studies included, twelve were conducted before the year 2000. Overall, 49 unique outcomes were measured in studies before 2000. In particular, the outcomes *thrombophlebitis*, *the acetabulum*, or *the weight-bearing dome* as well as *skin problems* were exclusively investigated in studies conducted before 2000.

*Heterotopic ossification*, which was investigated in eleven studies, and *quality of reduction*, which was investigated in nine studies, are the most frequently investigated outcomes in studies before the year 2000.

### Outcome names and definitions

There was a high variance in the terms used to name the same outcome. For example, for *heterotopic ossification*, the outcome terms varied enormously: “periarticular ossification,” “periarticular calcifications,” “paraarticular ossification,” “ectopic bone formation,” “ectopic ossification,” “heterotopic calcification,” “heterotopic bone formation,” “heterotrophic bone,” “ossifying myositis,” and “myositis ossificans.” Additionally, outcomes were defined heterogeneously or not at all. For example, *operation time* was assessed in 39 studies but defined in only eight studies (20.51%). Definitions ranged from “skin-to-skin” [27, 64, 71, 80, 163, 170, 191] to “[…] from reduction of the bone fragment to optimal placement of the internal fixation device” [204].

### Measurement time points

Measurement time points were often reported inconsistently. For example, in 48.57% of 70 studies that investigated the outcome *arthritis*, no measurement time points were given. Similarly, 50.00% of the 44 studies that examined *bone union* and 27.66% of the 47 studies that examined *radiologic outcomes* did not report time points.

In addition, many studies only reported “last follow-up” without providing further information on the exact time point. These included 18 studies investigating *radiologic outcomes*, five studies examining *bone union*, and eight studies reporting *arthritis*. Some studies described “postoperative” without further specification, including two studies on *radiologic outcomes*, two studies on *bone union*, and six studies on *arthritis*.

Additionally, several studies reported heterogeneous time points. In seven studies, *radiologic outcome* was examined at several time points; for example, follow-up was reported to be carried out at 6 and 12 weeks, 6 months, and annually postoperatively [141], while another study reported the follow-up at 45 days, 3, 6, and 12 months postoperative [26].

### Categorization

The included studies reported outcomes of all five core areas of the COMET taxonomy (*Death, Physiological/clinical, Life Impact, Resource use, Adverse events*) [22]. Overall, the studies assessed outcomes from 25 outcome domains (Table 2).

**Table 2:** Outcome categorization

Core area	Outcome domain	Number of unique outcomes	Example for unique outcomes
**Death**	Mortality/survival	1	Mortality
**Physiological/clinical**	Cardiac	1	Cardiovascular complications
	Gastrointestinal	6	Hernia
	General	16	pain
	Immune system	1	Inflammatory response
	Infection and infestation	7	Infection
	Injury and poisoning	3	Iatrogenic neurovascular injury
	Musculoskeletal and connective tissue	78	Range of motion
	Nervous system	25	Iatrogenic sciatic nerve injury
	Renal and urinary	5	Urinary tract infection
	Psychiatric	2	Mental status
	Respiratory, thoracic and mediastinal	3	Pneumonia
	Skin and subcutaneous tissue	13	Decubitus
	Vascular	23	Deep vein thrombosis
**Life impact**	Physical functioning	30	Ability to walk independently
	Social functioning	2	Social functioning
	Role functioning	4	Ability to work
	Emotional functioning/wellbeing	5	Arousal
	Cognitive functioning	1	Cognitive dysfunction
	Delivery of care	1	Satisfaction with medical care
**Resource use**	Economic	16	Operation time
	Hospital	5	Length of hospital stay
	Need for further intervention	12	Conversion to total hip arthroplasty
	Societal/carer burden	2	Return to their home
**Adverse events**	Adverse events/effects	4	Surgical complication

**Fig. 2:**
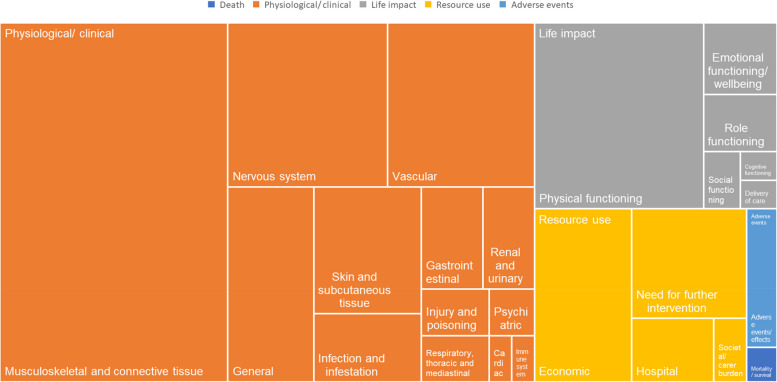
Proportion of outcomes in core domains

The most categorized outcome domains belong to the core area *Physiological/ clinical* (*n* = 13; 52.00%). Most of outcomes were related to the *Musculoskeletal and connective tissue* (*n* = 78; 29.32%), *Physical functioning* (*n* = 30; 11.28%), or *Nervous system* (*n* = 25; 9.40%) outcome domains. Only one outcome (0.38%) was categorized to the *Mortality/survival*, *Cardiac*, *Immune system*, *Cognitive functioning*, and *Delivery of care* outcome domains (Fig. 2).

**Fig. 3:**
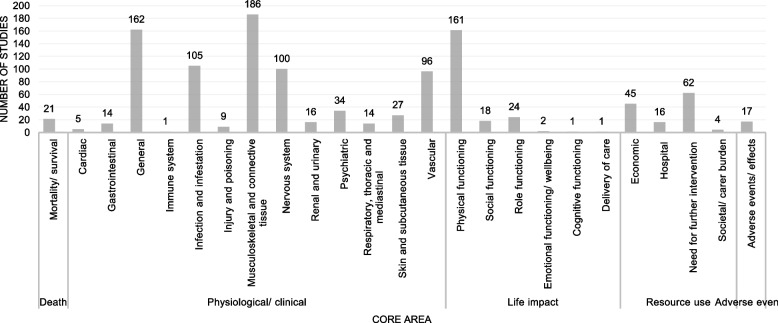
Number of studies reported outcomes from individual outcome domains

The number of studies that reported outcomes in the individual outcome domain is shown in Fig. 3. No outcome domain was considered in all 193 studies included. Most studies examined outcomes related to the *Musculoskeletal and connective tissue* (*n* = 186; 96.37%) outcome domain. Although only 16 of the 266 unique outcomes were assigned to the *General* outcome domain, it is still the second most frequently reported outcome domain (*n* = 162; 83.94%). Outcomes related to the *Immune system*, *Cognitive functioning*, and *Delivery of care* outcome domains were assessed by only one study. A complete table showing the categorization of all 266 unique outcomes is provided in Additional file 5.

### Measurement instruments

**Fig. 4:**
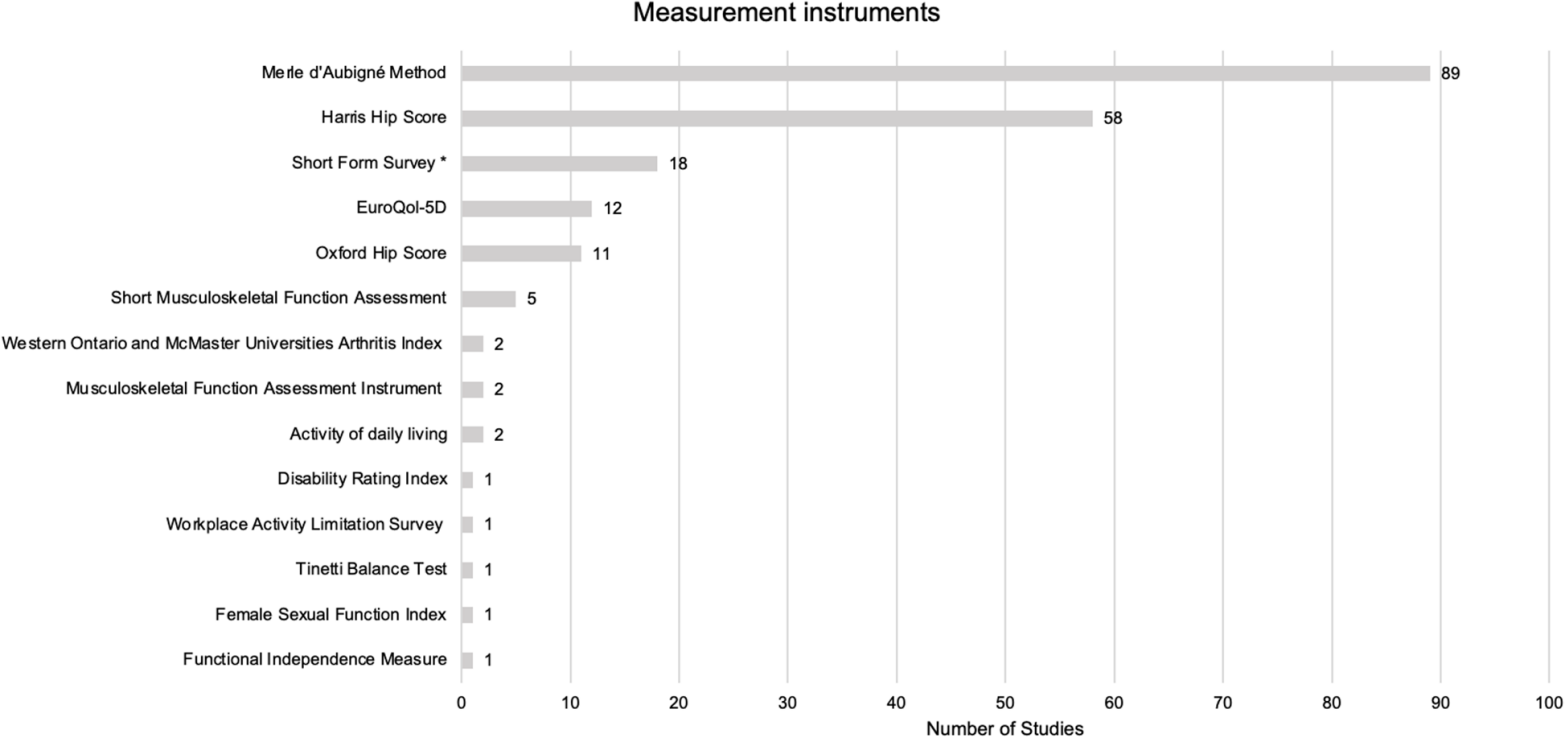
Measurement instruments. Legend: * Short Form Survey includes 8-Item Short Form Survey (SF-8), 12-Item Short Form Survey (SF-12), and 36-Item Short Form Survey (SF-36)

Overall, 17 different measurement instruments were used in 156 (80.83%) studies. The most frequently reported measurement instrument was the Merle d’Aubigné Method, which was used in 91 studies (58.33%). Five individual measurement instruments were utilized only in one study each. Figure 4 ranks the applied measurement instruments and displays the numbers of studies utilizing them.

Of the 156 studies, 28 (17.31%) used two measurement instruments, while 12 (7.69%) used a combination of three scores, such as the Harris Hip Score, the Merle d’Aubigné Method, and the Short Form Survey, to assess outcomes.

The 17 measurement instruments cover a spectrum of 50 unique outcomes. *Pain* was the most frequently assessed outcome. This outcome was reported in 153 studies (98.08%) and was assessed with nine different measurement instruments, including EuroQol-5D, Female Sexual Function Index, Harris Hip Score, Merle d’Aubigné Method, Oxford Hip Score, Short Form Survey, and Western Ontario and McMaster Universities Arthritis Index. Some of the studies utilized different measurement instruments, which partly included the same unique outcomes. For example, in 33 studies, two different measurement instruments, such as Merle d’Aubigné Method and Harris Hip Score [36, 47] or Oxford Hip Score and EuroQol-5D [70, 209, 214], were used which assessed *Pain*. This outcome was examined in 6 studies with three different measurement instruments. *Ability to walk independently* was the second most frequently assessed outcome and was measured in 136 studies (87.18%). Of these, 13 studies assessed this outcome using both the Merle d’Aubigné method and the Harris Hip Score.

*Range of motion* was reported in 134 studies (85.9%), with 55 studies using only the Harris Hip score and 85 studies using only the Merle d’Aubigné method. Eleven studies assessed *range of motion* with both instruments. A table of all measurement instruments including the number of studies that utilized them is provided in Additional file 6.

## Discussion

This systematic review shows that many different outcomes are measured in studies examining surgical treatment of acetabular fractures. None of the 266 unique outcomes were assessed in all 193 included studies. Five unique outcomes were measured in at least 60% of the studies, consisting of *pain*, *ability to walk independently*, *range of motion*, *quality of reduction*, and *heterotopic ossification*. Furthermore, almost 40% of the unique outcomes were measured in one study only. Many studies investigated specific aspects regarding the surgical treatment of acetabular fractures such as the effects on sexual function [119, 184]. The assessment of specific outcomes should not be hindered by a COS. Instead, studies should examine the most important outcomes defined in the future COS in addition to specific outcomes to reduce heterogeneity [12, 218]. Consequently, more study results could be included in evidence synthesis like in systematic reviews or clinical guidelines, which would increase statistical power and lead to more precise findings and, thus, improve the knowledge generated from research [15, 219]. This is particularly important in studies investigating the surgical treatment of acetabular fractures as this is a rare fracture, and studies can therefore only investigate a small number of individuals [1–3].

In addition, this systematic review highlights significant inconsistencies in the reporting of measurement time points across studies assessing surgical treatment of acetabular fractures. Frequently, measurement time points were not specified and vague time points, such as “last follow-up” or “postoperative” given without further specification. Moreover, the studies showed considerable variability of time points with some studies reporting outcomes at multiple heterogeneous time points. This is comparable with the systematic review by Copley et al. (2022), in which only 56.7% of the included studies on cervical spine fractures reported a precise measurement time point [220]. Moreover, the vast variability in measurement instruments used across studies also presents a challenge for synthesizing study results as the different measurement instruments contain various scales and items. Other systematic reviews examining outcomes assessed in studies on traumatology also identified a high number of heterogenous measurement instruments used [221–225]. Overall, there is a need for predefined, standardized measurement time points and the use of measurement instruments in future research to ensure consistency and to improve the comparability of findings across studies.

Outcomes on the *musculoskeletal and connective tissue* outcome domain were reported in the majority (96.37%) of included studies. This is comparable with other systematic reviews in traumatological research; for example, a systematic review for the development of a COS for traumatic brachial plexus injuries showed that outcomes from this domain were reported most often with 86% of included studies [222]. Similarly, Aquilina et al. (2023) showed that most of the outcomes reported in studies on open lower limb fractures related to the *musculoskeletal and connective tissue* outcome domain [221].

This study was characterized by a clear and reproducible approach based on published guidelines and the classification of outcomes according to the COMET taxonomy [12, 22]. In addition, this systematic review was conducted by reviewers with methodological or clinical expertise which enabled multidisciplinary approaches. The consideration of published and ongoing studies made it possible to identify contemporary outcomes. No publication date restrictions were defined in the inclusion criteria which made it possible to analyze a high number of studies and outcomes as well as the differences between studies published before and after 2000; however, the analysis showed no differences in the reported outcomes in studies conducted before and after the year 2000.

In consideration of these advantages, some limitations are noted. Only studies in German and English were included. Nevertheless, the comprehensive and sensitive literature search on three databases and two study registries without temporal or geographical restrictions reduced the risk of missing relevant studies. Also, broad inclusion criteria were defined to identify a high number of relevant outcomes for studies examining surgical treatment of acetabular fractures. Therefore, with 193 included studies, this systematic review is the most comprehensive examination of outcome reporting in studies examining acetabular fractures.

## Conclusion

This systematic review highlights the absence of standardized methodologies for assessing and reporting outcomes in studies that investigate surgical treatment of acetabular fractures. This significantly limits the ability to synthesize and compare the results of these studies.

This systematic review provides the basis for the development of a COS which can reduce the heterogeneity of outcomes in and between future studies. In a subsequent study, the Delphi method will be used to develop the COS for surgically treated acetabular fractures. This will help to ensure standardized outcome assessment in future studies, reduce heterogeneity between studies, and thereby enhance more study results to be included in evidence synthesis, and thus improve knowledge generated from research.

## Supplementary Information

Additional file 1. PRISMA checklist.

Additional file 2. COS-STAR checklist.

Additional file 3. Search strategies.

Additional file 4. Study characteristics.

Additional file 5. Outcome categorization.

Additional file 6. Measurement instruments.

## Data Availability

The datasets used and/or analyzed during the current study are available from the corresponding author on reasonable request.

## Abbreviations

CENTRAL: Cochrane Central Register of Controlled Trials
COMET: Core Outcome Measures in Effectiveness Trials
COS: Core outcome set
COS-STAR: Core Outcome Set-STAndards for Reporting
ORBIT: Outcome reporting bias in trials
PRISMA: Preferred Reporting Items for Systematic Reviews and Meta-Analyses
RCT: Randomized controlled trial
