# Supplementary material for: Developing a core outcome set for acetabular fractures: a systematic review (part I)
Source: Syst Rev. 2025 Apr 9;14:83. doi: 10.1186/s13643-025-02824-0 (PMC11983908; doi:10.1186/s13643-025-02824-0)
Supplement: Supplementary file 2 — Additional file 2. COS-STAR checklist. [file 13643_2025_2824_MOESM2_ESM.docx]

**COS-STAR checklist**

| **SECTION/TOPIC** | **ITEM No.** | **CHECKLIST ITEM** | **REPORTED ON PAGE NUMBER** |
| --- | --- | --- | --- |
| TITLE/ABSTRACT | | | |
| Title | 1a | Identify in the title that the paper reports the development of a COS | p. 1 |
| Abstract | 1b | Provide a structured summary | p. 2 |
| INTRODUCTION | | | |
| Background and Objectives | 2a | Describe the background and explain the rationale for developing the COS. | p. 4 -5 |
|  | 2b | Describe the specific objectives with reference to developing a COS. | p. 5 |
| Scope | 3a | Describe the health condition(s) and population(s) covered by the COS. | p. 4 |
|  | 3b | Describe the intervention(s) covered by the COS. | p. 4 -5 |
|  | 3c | Describe the setting(s) in which the COS is to be applied. | p. 5 |
| METHODS | | | |
| Protocol/Registry Entry | 4 | Indicate where the COS development protocol can be accessed, if available, and/or the study registration details. | p. 6 |
| Participants | 5 | Describe the rationale for stakeholder groups involved in the COS development process, eligibility criteria for participants from each group, and a description of how the individuals involved were identified. | Not applicable |
| Information Sources | 6a | Describe the information sources used to identify an initial list of outcomes. | p. 6 |
|  | 6b | Describe how outcomes were dropped/combined, with reasons (if applicable). | p. 6 -7 |
| Consensus Process | 7 | Describe how the consensus process was undertaken. | Not applicable |
| Outcome Scoring | 8 | Describe how outcomes were scored and how scores were summarised. | Not applicable |
| Consensus Definition | 9a | Describe the consensus definition. | Not applicable |
|  | 9b | Describe the procedure for determining how outcomes were included or excluded from consideration during the consensus process. | Not applicable |
| Ethics and Consent | 10 | Provide a statement regarding the ethics and consent issues for the study. | Not applicable |
| RESULTS | | | |
| Protocol Deviations | 11 | Describe any changes from the protocol (if applicable), with reasons, and describe what impact these changes have on the results. | Not applicable |
| Participants | 12 | Present data on the number and relevant characteristics of the people involved at all stages of COS development. | Not applicable |
| Outcomes | 13a | List all outcomes considered at the start of the consensus process. | p. 68 -70 |
|  | 13b | Describe any new outcomes introduced and any outcomes dropped, with reasons, during the consensus process. | Not applicable |
| COS | 14 | List the outcomes in the final COS. | Not applicable |
| DISCUSSION | | | |
| Limitations | 15 | Discuss any limitations in the COS development process. | p. 15 -16 |
| Conclusions | 16 | Provide an interpretation of the final COS in the context of other evidence, and implications for future research. | Not applicable |
| OTHER INFORMATION | | | |
| Funding | 17 | Describe sources of funding/role of funders. | p. 17 |
| Conflicts of Interest | 18 | Describe any conflicts of interest within the study team and how these were managed. | p. 17 |

*From: Kirkham JJ, Gorst S, Altman DG, Blazeby JM, Clarke M, Devane D, et al. (2016) Core Outcome Set–STAndards for Reporting: The COS-STAR Statement. PLoS Med 13(10): e1002148. https://doi.org/10.1371/journal.pmed.1002148*
