# Supplementary material for: Developing a core outcome set for acetabular fractures: a systematic review (part I)
Source: Syst Rev. 2025 Apr 9;14:83. doi: 10.1186/s13643-025-02824-0 (PMC11983908; doi:10.1186/s13643-025-02824-0)
Supplement: Supplementary file 3 — Additional file 3. Search strategies. [file 13643_2025_2824_MOESM3_ESM.docx]

**Search strategies**

**MEDLINE via PubMed**:

1. (acetabul*[Title/Abstract]) OR (acetabulum[MeSH Terms])

2. (fracture*[Title/Abstract]) OR (fracture, bone[MeSH Terms])

3. #1 AND #2

**CENTRAL**:

#1 MeSH descriptor: [Acetabulum] explode all trees

#2 (acetabul*):ti,ab,kw

#3 (fracture*):ti,ab,kw

#4 MeSH descriptor: [Fracture Fixation] explode all trees

#5 #1 OR #2

#6 #3 OR #4

#7 #5 AND #6

**Web of Science Core Collection:**

(TS=(acetabul*)) AND TS=(fracture*)

**Clinicaltrials.gov**:

(acetabular OR acetabulum) AND (fracture OR fractures)

**WHO**:

(acetabular OR acetabulum) AND (fracture OR fractures)
