# Supplementary material for: Developing a core outcome set for acetabular fractures: a systematic review (part I)
Source: Syst Rev. 2025 Apr 9;14:83. doi: 10.1186/s13643-025-02824-0 (PMC11983908; doi:10.1186/s13643-025-02824-0)
Supplement: Supplementary file 4 — Additional file 4. Study characteristics. [file 13643_2025_2824_MOESM4_ESM.docx]

**Additional file 4. Study characteristics**

| **Author (year) study reference** | **Title** | **Study design** | **Country** | **Number of patients** |
| --- | --- | --- | --- | --- |
| Aigner et al. (2017)​ [23]​ | Internal fixation of acetabular fractures in an older population using the TIMI approach – Midterm results of a prospective study | OS without control group | Germany | 47 |
| Al Adawy et al. (2020) ​[24]​ | Modified Stoppa as an alternative surgical approach for fixation of anterior fracture acetabulum: a randomized control clinical trial | RCT | Egypt | 38 |
| Alexa et al. (2019) ​[26]​ | OUR EXPERIENCE IN THE SURGICAL TREATMENT OF ACETABULAR FRACTURES USING “SPRING PLATE” TECHNIQUE | OS without control group | Romania | 21 |
| Amoretti et al. (2013) ​[25]​ | Percutaneous Screw Fixation of Acetabular Roof Fractures by Radiologists Under CT and Fluoroscopy Guidance | OS without control group | France | 52 |
| Ansari et al. (2021) ​[27]​ | Role of 3D printing in the management of complex acetabular fractures: a comparative study | OS with control group | India | 27 |
| Askam, & Sims (2019) ​[28]​ | Supplemental Superior Buttress Plating for the Treatment of Posterosuperior Wall Acetabulum Fractures | OS with control group | USA | 31 |
| Baba, & Shitoto (2010) ​[29]​ | Cable Wxation of acetabular fracture utilizing an anchor screw for reduction and fixation | OS without control group | Japan | 10 |
| Bogdan et al. (2015) ​[30]​ | Neurologic Injury in Operatively Treated Acetabular Fractures | OS without control group | USA | 137 |
| Borens et al. (2004) ​[31]​ | Die Behandlung von Acetabulumfrakturen bei geriatrischen Patienten mittels modifizierter Kabelcerclage und primärer Hüfttotalprothese [Treatment of acetabular fractures in the elderly with primary total hip arthroplasty and modified cerclage. Early results] | OS without control group | Switzerland | 15 |
| Bouabdellah et al. (2022) ​[32]​ | Advantages of threedimensional printing in the management of acetabular fracture fixed by the KocherLangenbeck approach: randomised controlled trial | RCT | Tunisia | 43 |
| Butler et al. (2022) ​[33]​ | The Effect of Surgeon Experience on Short- to Medium-Term Complication Rate Following Operative Fixation of Acetabular Fractures | OS without control group | USA | 795 |
| Cano-Luís et al. (2023) ​[34]​ | Pararectus approach in acetabular fractures in patients older than 65 years. Is it possible to improve the technique? | OS without control group | Spain | 46 |
| Carrothers, & Alvarez-Berdugo; ISRCTN16739011 (ongoing) ​[209]​ | AceFIT – a study comparing three methods of treatment of acetabular fractures (a type of hip fracture) in older patients; surgical fixation versus surgical fixation and hip replacement versus non-surgical treatment | RCT | UK | *Target: 60* |
| Caterini et al. (2000) ​[35]​ | Immediate passive mobilization of the hip after internal fixation of acetabular fractures | OS without control group | Italy | 16 |
| Cavalié et al. (2022) ​[36]​ | Clinical and radiological outcomes of antegrade posterior column screw fixation of the acetabulum | OS with control group | France | 69 |
| Ceylan et al. (2014) ​[38]​ | External Rotator Sparing with Posterior Acetabular Fracture Surgery: Does It Change Outcome? | OS without control group | Turkey | 20 |
| Chen et al. (2000) ​[37]​ | Treatment of acetabular fractures: 10-year experience | OS with control group | China | 73 |
| Chen et al. (2019) ​[42]​ | A combination of the modified Stoppa approach and the iliac fossa approach in treating compound acetabular fractures by using an anterior ilioischial plate | OS with control group | China | 40 |
| Chen et al. (2021) ​[40]​ | Trochanteric osteotomy for acetabular fracture fixation: a case series and literature review | OS without control group | USA | 26 |
| Chen et al. (2022) ​[41]​ | Oblique-ilioischial plate technique: a novel method for acetabular fractures involving low posterior column | OS without control group | China | 18 |
| Chen et al. (2022) ​[39]​ | One-stop computerized virtual planning system for the surgical management of posterior wall acetabular fractures | OS with control group | China | 52 |
| Chiu et al. (1996) ​[44]​ | Fractures of posterior wall of acetabulum | OS with control group | China | 27 |
| Chiu et al. (2000) ​[43]​ | Surgical treatment of displaced acetabular fractures - 72 cases followed for 10 (6±14) years | OS without control group | Taiwan | 72 |
| Ciolli et al. (2021) ​[45]​ | Anterior intrapelvic approach and suprapectineal quadrilateral surface plate for acetabular fractures with anterior involvement: a retrospective study of 34 patients | OS without control group | Italy | 34 |
| Crowl, & Kahler (2002) ​[46]​ | Closed Reduction and Percutaneous Fixation of Anterior Column Acetabular Fractures | OS without control group | USA | 23 |
| Dadura et al. (2022) ​[47]​ | Radiological and Functional Assessment of Treatment Outcomes in Patients after Open Reduction with Internal Fixation (ORIF) of Acetabular Fractures | OS without control group | Poland | 31 |
| Dickinson et al. (1993) ​[49]​ | Muscle Strength Testing Following Surgery for Acetabular Fractures | OS without control group | USA | 30 |
| Ebraheim et al. (2007) ​[48]​ | Sliding trochanteric osteotomy in acetabular fractures: A review of 30 cases | OS without control group | USA | 30 |
| Eckardt et al. (2015) ​[50]​ | Open reduction and internal fixation aided by intraoperative 3-dimensional imaging improved the articular reduction in 72 displaced acetabular fractures | OS with control group | Denmark | 114 |
| Elmadağ et al. (2014) ​[51]​ | The Stoppa approach versus the ilioinguinal approach for anterior acetabular fractures: A case control study assessing blood loss complications and function outcomes | OS with control group | Turkey | 36 |
| Enocson, & Blomfeldt (2014) ​[53]​ | Acetabular Fractures in the Elderly Treated With a Primary Burch–Schneider Reinforcement Ring, Autologous Bone Graft, and a Total Hip Arthroplasty: A Prospective Study With a 4-Year Follow-Up | OS without control group | Sweden | 15 |
| Erem et al. (2019) ​[52]​ | Effects of the Incision Preference in Acetabular Surgery on the Postoperative Functional Outcomes | OS with control group | Turkey | 47 |
| Ernstberger et al. (2021) ​[55]​ | Minimally displaced acetabulum fractures in geriatric patients: a comparison of open, percutaneous and nonoperative treatment from the German Pelvic Injury Register data | OS with control group | Germany | 179 |
| Etemadifar et al. (2016) ​[54]​ | Operative management of acetabular fracture: A 10year experience in Isfahan, Iran | OS without control group | Iran | 30 |
| Fahmy et al. (2018) ​[58]​ | Single Versus Double Column Fixation in Transverse Fractures of the Acetabulum: A Randomised Controlled Trial | RCT | Egypt | 30 |
| Fakru et al. (2021) ​[57]​ | Functional Outcome of Surgical Stabilisation of Acetabular Fractures | OS without control group | Malaysia | 41 |
| Firoozabadi et al. 2020) ​[56]​ | Isolated Buttress Plating of Posterior Wall Acetabular Fractures: Is it Sufficient? | OS with control group | USA | 101 |
| Frietman et al. (2018) ​[61]​ | Patient-reported outcome measures after surgery for an acetabular fracture | OS without control group | Netherlands | 220 |
| Gary et al. (2011) ​[60, 207]​ | Survivorship of the native hip joint after percutaneous repair of acetabular fractures in the elderly & Functional outcomes in elderly patients with acetabular fractures treated with minimally invasive reduction and percutaneous fixation (2. report) | OS without control group | USA | 75 |
| Giannoudis et al. (2009) ​[68]​ | Factors determining quality of life and level of sporting activity after internal fixation of an isolated acetabular fracture | OS without control group | UK | 52 |
| Giannoudis et al. (2013) ​[59]​ | Acetabular fractures with marginal impaction MID-TERM RESULTS | OS without control group | UK, & Greece | 60 |
| Gorczyca et al. (1995) ​[67]​ | Lateral extension of the ilioinguinal incision in the operative treatment of acetabulum fractures | OS without control group | Canada | 26 |
| Gültaç et al. (2019) ​[66]​ | Surgical treatment of acetabulum posterior wall fractures: Comparison between undercountering and marginal impaction reconstruction method with odd methods | OS with control group | Turkey | 21 |
| Gültaç et al. (2022) ​[69]​ | A device that facilitates screwing at an appropriate angle in quadrilateral surface fractures: 105-degree drill attachment | OS without control group | Turkey | 35 |
| Gupta et al. (2017) ​[65]​ | The role of trochanteric flip osteotomy in fixation of certain acetabular fractures | OS without control group | India | 25 |
| Gusic et al. (2015) ​[64]​ | Rationale for more consistent choice of surgical approaches for acetabular fractures | OS with control group | Croatia | 156 |
| Güven et al. (2018) ​[63]​ | The outcomes of acetabular fractures treated surgically and factors affecting the result The results of acetabular fractures | OS without control group | Turkey | 30 |
| Hammad et al. (2017) ​[62]​ | The efficacy of posterior plating and anterior column screw fixation in the management of T-shaped acetabular fractures - CART analysis of prospective cohort study | OS without control group | Egypt | 34 |
| Hao et al. (2016) ​[74]​ | Temporary Balloon Occlusion of the Abdominal Aorta in Treatment of Complex Acetabular Fracture | OS with control group | China | 41 |
| Heck et al. (1997) ​[73]​ | Direct complications of trochanteric osteotomy in open reduction and internal fixation of acetabular fractures | OS without control group | USA | 55 |
| Heeg et al. (1990) ​[72]​ | Operative treatment for acetabular fractures | OS without control group | Netherlands | 54 |
| Henawy, & Atef; PACTR202204468763535 (ongoing) ​[210]​ | Assessment of Modified Kocher-Langenbeck Surgical Approach Compared with Conventional Kocher-Langenbeck Surgical Approach in Treatment of Simple Posterior Wall Acetabular Fractures | RCT | Egypt | *Target: 36* |
| Herscovici et al. (2010) ​[71]​ | The Combined Hip Procedure: Open Reduction Internal Fixation Combined With Total Hip Arthroplasty for the Management of Acetabular Fractures in the Elderly | OS without control group | USA | 22 |
| Hislop et al. (2022) ​[70]​ | Fix and replace: Simultaneous fracture fixation and hip replacement for acetabular fractures in older patients | OS without control group | UK | 57 |
| Hou, & Shang;  NCT02327949 (ongoing) ​[212]​ | Prospective Comparison Between W-Shaped Acetabular Angular Plate (WAAP) and Reconstruction Plate for the Treatment of Posterior Acetabular Wall Fracture | RCT | China | *Target: 40* |
| Hou;  NCT03193840 (ongoing) ​[211]​ | Surgical Treatment of Old Acetabular Fracture With Posterior Wall Osteotomy | OS without control group | China | *Target: 40* |
| Huang et al. (2020) ​[80]​ | Surgical treatment for both-column acetabular fractures using pre-operative virtual simulation and three-dimensional printing techniques | RCT | China | 40 |
| Huda et al. (2021) ​[81]​ | Factors affecting the functional outcome of surgically managed displaced acetabular fractures | OS without control group | India | 50 |
| Inoue et al. (2021) ​[79]​ | Osteosynthesis for Geriatric Acetabular Fractures: An Epidemiological and Clinico-Radiological Study Related to Marginal or Roof Impaction | OS without control group | Japan | 68 |
| Iqbal et al. (2016) ​[78]​ | Functional and Radiological Outcome of Surgical Management of Acetabular Fractures in Tertiary Care Hospital | OS without control group | Pakistan | 50 |
| Iqbal et al. (2017) ​[77]​ | Surgical outcomes of acute acetabular transverse fracture using ilioinguinal and Stoppa approach | OS with control group | Pakistan | 65 |
| Islam et al. (2020) ​[76]​ | Outcome of Open Reduction and Internal Fixation of Posterior Wall Fracture of Acetabulum | OS without control group | Bangladesh | 25 |
| Jouffroy;  NCT03312491 (ongoing) ​[213]​ | Treatment of Acetabular Fracture: the Contribution of the 3D Impression | OS without control group | France | *Target: 30* |
| Kang, & Min (2002) ​[75]​ | Cable fixation in displaced fractures of the acetabulum: 21 patients followed for 2-8 years | OS without control group | Korea | 21 |
| Kim et al. (2014) ​[85]​ | The submuscular sliding plate technique for acetabular posterior wall fractures extending to the acetabular roof | OS without control group | South Korea | 13 |
| Kim et al. (2015) ​[84]​ | Modified Stoppa Approach for Surgical Treatment of Acetabular Fracture | OS without control group | Korea | 22 |
| Kim et al. (2017) ​[89]​ | Injury of the obturator nerve in the modified Stoppa approach for acetabular fractures | OS with control group | Korea | 22 |
| Kim et al. (2018) ​[86]​ | Effectiveness of Hip Arthroscopy Performed Simultaneously before Open Reduction and Internal Fixation for Acetabular Fracture and Fracture-dislocation of the Hip | OS with control group | Korea | 54 |
| Kubota et al. (2012) ​[88]​ | Changes in Gait Pattern and Hip Muscle Strength After Open Reduction and Internal Fixation of Acetabular Fracture | OS with control group | Japan | 19 |
| Kumar et al. (2021) ​[83, 208]​ | Outcome of acetabulum fractures treated with open reduction and internal fixation through Kocher-Langenbeck Approach: A retrospective study  &  Outcome of total hip arthroplasty in patients with failed open reduction and internal fixation of acetabular fractures (2. report) | OS without control group | India | 80 |
| Lannes et al. (2020) ​[87]​ | Outcomes of combined hip procedure with dual mobility cup versus osteosynthesis for acetabular fractures in elderly patients: a retrospective observational cohort study of fifty one patients | OS with control group | Switzerland | 51 |
| Lawyer et al. (2014) ​[82]​ | Prevalence of Post-Traumatic Osteoarthritis in Morbidly Obese Patients after Acetabular Fracture Fixation | OS with control group | USA | 39 |
| Lee, & Johnson (2018) ​[93]​ | Use of Spring Plates in Fixation of Comminuted Posterior Wall Acetabular Fractures | OS without control group | USA | 52 |
| Li et al. (2014) ​[95]​ | Fractures of the posterior wall of the acetabulum: Treatment using internal fixation of two parallel reconstruction plates | OS without control group | China | 57 |
| Li et al. (2022) ​[94]​ | Midterm results of digastric trochanteric flip osteotomy for high acetabular posterior wall fracture | OS with control group | China | 39 |
| Liaw et al. (2022) ​[92]​ | Primary total hip replacement using Burch-Schneider cages for acetabular fractures | OS without control group | UK | 12 |
| Lin et al. (2015) ​[97]​ | Functional Outcomes After Total Hip Arthroplasty for the Acute Management of Acetabular Fractures: 1- to 14-Year Follow-up | OS without control group | USA | 33 |
| Liu et al. (2022) ​[96]​ | The Kocher-Langenbeck approach combined with robot-aided percutaneous anterior column screw fixation for transverse-oriented acetabular fractures: a retrospective study | OS with control group | China | 29 |
| Long et al. (2017) ​[91]​ | Effects of the acetabular fracture index and other factors of posterior wall acetabular fracture on functional outcome | OS with control group | China | 48 |
| Lont et al. (2019) ​[99]​ | Total hip arthroplasty, combined with a reinforcement ring and posterior column plating for acetabular fractures in elderly patients: good outcome in 34 patients | OS with control group | Finland | 59 |
| Lovrić et al. (2011) ​[98]​ | Influence of Surgery onto the Appearance of the Hip Joint Periarticular Calcification in Patients with the Acetabular Fracture | OS with control group | Croatia | 21 |
| Lubovsky et al. (2013) ​[90]​ | Quantitative Measures of Damage to Subchondral Bone Are Associated With Functional Outcome Following Treatment of Displaced Acetabular Fractures | OS without control group | Canada | 22 |
| Lundin et al. (2023) ​[103]​ | Complications after surgical treatment of acetabular fractures: a 5year followup of 229 patients | OS with control group | Sweden | 229 |
| MacCormick et al. (2019) ​[104]​ | Acute total hip arthroplasty versus open reduction internal fixation for posterior wall acetabular fractures in middle-aged patients | OS with control group | USA | 48 |
| Magill et al. (2012) ​[102]​ | Minimum ten-year follow-up of acetabular fracture fixation from the Irish tertiary referral centre | OS without control group | Ireland | 21 |
| Magu et al. (2014) ​[105]​ | Long term results after surgical management of posterior wall acetabular fractures | OS without control group | India | 25 |
| Magu et al. (2016) ​[106]​ | Modified Kocher-Langenbeck approach is combined surgical exposures for acetabular fractures management | OS without control group | India | 20 |
| Maini et al. (2014) ​[101]​ | Surgical dislocation of the hip for reduction of acetabular fracture and evaluation of chondral damage | OS without control group | India | 22 |
| Malhotra et al. (2013) ​[107]​ | Acute Total Hip Arthroplasty in Acetabular Fractures in the Elderly Using the Octopus System Mid Term to Long Term Follow-Up | OS without control group | India | 15 |
| Manson et al. (2022) ​[100]​ | Open reduction and internal fixation alone versus open reduction and internal fixation plus total hip arthroplasty for displaced acetabular fractures in patients older than 60 years: A prospective clinical trial | Mixed methods study (RCT & OS with control group) | USA | 47 |
| Manzoor et al. (2021) ​[108]​ | Osteosynthesis of Common Acetabular Fractures Operated on Through a Single Posterior (KocherLangenbeck) Approach with or without Trochanteric Flip Osteotomy. A Case Series | OS without control group | India | 42 |
| Mardani-Kivi et al. (2013) ​[110]​ | Surgical Treatment of acetabular fractures and its learning curve | OS with control group | Iran | 104 |
| Märdian et al. (2015) ​[109]​ | Fixation of acetabular fractures via the ilioinguinal versus pararectus approach A DIRECT COMPARISON | OS with control group | Germany | 100 |
| Masse et al. (2013) ​[113]​ | Surgical Dislocation Technique for the Treatment of Acetabular Fractures | OS without control group | Italy | 31 |
| Matta et al. (1986) ​[112]​ | Fractures of the acetabulum - A retrospective analysis | OS with control group | USA | 43 |
| McDowell et al. (2012) ​[111]​ | Modified Ollier Transtrochanteric Approach for the Treatment of Acetabular Fractures | OS without control group | USA | 94 |
| McGee et al. (2023) ​[116]​ | Preoperative Blood Loss of Isolated Acetabular Fractures | OS with control group | USA | 598 |
| McMahon et al. (2020) ​[115]​ | Coned hemipelvis reconstruction for osteoporotic acetabular fractures in frail elderly patients - minimum one-year follow-up | OS without control group | UK | 21 |
| Meena et al. (2022) ​[114]​ | Treatment of acetabular fractures with quadrilateral plate injury – a comparison of two commonly used methods | OS with control group | India | 90 |
| Melo, & Khoshbin;  NCT03713853 (ongoing) ​[214]​ | Geriatric Acetabular Fractures: Open Reduction Internal Fixation Versus Replacement - A Large Cohort of Acute Open Reduction Internal Fixation (ORIF) Versus Total Hip Arthroplasty for Geriatric Acetabular Fractures | OS with control group | Canada | *Target: 104* |
| Mesbahi et al. (2018) ​[121]​ | Outcome after Surgical Management of Acetabular Fractures: A 7-Year Experience | OS without control group | Iran | 79 |
| Miller et al. (2010) ​[122]​ | The radiological evaluation of acetabular fractures in the elderly | OS without control group | USA | 45 |
| Min et al. (2018) ​[120]​ | Outcomes are equivalent for two-column acetabular fractures either with or without posterior-wall fractures | OS with control group | South Korea | 42 |
| Mitsionis et al. (2012) ​[123]​ | Surgical Management of Posterior Hip Dislocations Associated With Posterior Wall Acetabular Fracture: A Study With a Minimum Follow-Up of 15 Years | OS without control group | Greece | 19 |
| Moed et al. (2014) ​[124]​ | The modified Gibson approach to the acetabulum | OS without control group | USA | 16 |
| Monteleone et al. (2023) ​[119]​ | Quality of life from return to work and sports activities to sexual dysfunction after surgical treatment of acetabular fractures | OS without control group | Switzerland | 65 |
| Moroni et al. (1995) ​[125]​ | Surgical treatment of both-column fractures by staged combined ilioinguinal and Kocher-Langenbeck approaches | OS without control group | Italy | 18 |
| Mouhsine et al. (2002) ​[118]​ | Acute total hip arthroplasty for acetabular fractures in the elderly 11 patients followed for 2 years | OS without control group | Switzerland | 11 |
| Mouhsine et al. (2004) ​[126]​ | Cable Fixation and Early Total Hip Arthroplasty in the Treatment of Acetabular Fractures in Elderly Patients | OS without control group | Switzerland | 18 |
| Mouhsine et al. (2005) ​[127]​ | Percutaneous retrograde screwing for stabilisation of acetabular fractures | OS without control group | Switzerland | 18 |
| Muzii et al. (2021) ​[117]​ | Radiographic and functional outcome of complex acetabular fractures: implications of open reduction in spinopelvic balance, gait and quality of life | OS with control group | Italy | 26 |
| Naranje et al. (2010) ​[130]​ | Digastric trochanteric filp osteotomy and surgical dislocation of hip in the management of acetabular fractures | OS without control group | India | 18 |
| Nayak et al. (2020) ​[131]​ | Short-term results of surgical treatment of acetabular fractures using the modified Stoppa approach | OS without control group | India | 23 |
| Negrin et al. (2010) ​[129]​ | Prone or Lateral? Use of the Kocher-Langenbeck Approach to Treat Acetabular Fractures | OS with control group | Austria | 104 |
| Negrin, & Seligson (2010) ​[128]​ | The Kocher-Langenbeck Approach: Differences in Outcome of Transverse Acetabular Fractures Depending on the Patient’s Position | OS with control group | Austria | 27 |
| Nicol et al. (2020) ​[132]​ | Does use of a quadrilateral surface plate improve outcome in elderly acetabular fractures? | OS with control group | Canada | 30 |
| Nicol et al. (2021) ​[143]​ | Outcomes of Total Hip Arthroplasty After Acetabular Open  Reduction and Internal Fixation in the Elderly - Acute vs Delayed Total Hip Arthroplasty | OS with control group | Canada | 26 |
| Oh et al. (2006) ​[142]​ | Results after operative treatment of transverse acetabular fractures | OS without control group | Korea | 15 |
| Ortega-Briones et al. (2017) [141]​ | Acetabular Fractures in the Elderly: Midterm Outcomes of  Column Stabilisation and Primary Arthroplasty | OS without control group | UK | 13 |
| O'Toole et al. (2014) ​[140]​ | How Often Does Open Reduction and Internal Fixation of Geriatric Acetabular Fractures Lead to Hip Arthroplasty? | OS with control group | USA | 52 |
| Øvre et al. (2008) ​[139]​ | Acetabular fracture displacement, roof arc angles and 2 years outcome | OS with control group | Norway | 107 |
| Pantazopoulos et al. (1993) ​[144]​ | Surgical treatment of acetabular posterior wall fractures | OS without control group | Greece | 52 |
| Park et al. (2017) ​[138]​ | Outcome of alternative approach to displaced acetabular fractures | OS without control group | South Korea | 23 |
| Patil et al. (2021) ​[137]​ | A Single Approach for Management of Fractures Involving Both Columns of the Acetabulum: A Case Series of 23 Patients | OS without control group | India | 23 |
| Patterson et al. (2023) ​[145]​ | Early hip survival after open reduction internal fixation of acetabular fracture | OS without control group | USA | 852 |
| Perumal et al. (2020) ​[136]​ | Marginal impaction in complex posterior wall acetabular fractures: role of allograft and midterm results | OS without control group | India | 16 |
| Peter (2015) ​[135]​ | Open reduction and internal fixation of osteoporotic acetabular fractures through the ilio-inguinal approach: use of buttress plates to control medial displacement of the quadrilateral surface | OS without control group | Switzerland | 13 |
| Pillella, & John (2020) ​[134]​ | A Prospective Study on Functional Outcome of Open Reduction and Internal Fixation of Acetabular Fractures | OS without control group | India | 30 |
| Pompili et al. (2012) ​[133]​ | Surgical treatment of displaced acetabular fractures: report of 13 clinical cases | OS without control group | Italy | 13 |
| Preston et al. (2021) ​[151]​ | Survivorship of the Hip Joint After Acetabulum Fracture | OS without control group | USA | 417 |
| Qadir, & Bukhari (2015) ​[150]​ | OUTCOME OF OPERATIVE TREATMENT OF ACETABULAR FRACTURES: SHORT TERM FOLLOW-UP | OS without control group | Pakistan | 19 |
| Qi et al. (2009) ​[149]​ | Treatment of posterior wall fractures of acetabulum | OS without control group | China | 31 |
| Ragnarsson et al. (1992) ​[152]​ | The triradiate incision for acetabular fractures - A prospective study of 23 cases | OS without control group | Sweden | 23 |
| Rahimi et al. (2013) ​[148]​ | Surgical Management of Acetabular Fractures: A Case Series | OS without control group | Iran | 44 |
| Rickman et al. (2014) ​[153]​ | Managing Acetabular Fractures in the Elderly With Fixation and Primary Arthroplasty - Aiming for Early Weightbearing | OS without control group | UK | 24 |
| Roetman et al. (2006) ​[147]​ | Langzeitergebnisse nach Azetabulumfrakturen unter Berücksichtigung von heterotopen Ossifikationen [Long-Term Results after Acetabular Fractures with Respect to Heterotopic Ossifications] | OS with control group | Germany | 55 |
| Rommens et al. (2001) ​[154]​ | Posterior Wall Fractures of the Acetabulum: Characteristics, Management, Prognosis | OS without control group | Germany | 46 |
| Rommens et al. (2020) ​[156]​ | Open reduction and internal fixation of acetabular fractures in patients of old age | OS with control group | Germany | 26 |
| Rommens et al. (2020) ​[146]​ | Medial buttressing of the quadrilateral surface in acetabular and periprosthetic acetabular fractures | OS with control group | Germany | 46 |
| Ruktrakul;  TCTR20201222001 (ongoing) ​[215]​ | Clinical results after fixation of acetabular fracture via Pararectus approach versus the modified Stoppa: Randomized controlled trial | RCT | Thailand | *Target: 46* |
| Salama et al. (2017) ​[155]​ | Simultaneous open reduction and internal fixation and total hip arthroplasty for the treatment of osteoporotic acetabular fractures | OS without control group | Egypt | 18 |
| Salameh et al. (2021) ​[163]​ | The role of patient positioning on the outcome of acetabular fractures fixation through the Kocher–Langenbeck approach | OS with control group | Qatar | 73 |
| Salar et al. (2017) ​[162]​ | Total hip arthroplasty for acetabular fractures: “Early Application” | OS without control group | Turkey | 17 |
| Sarantis et al. (2020) ​[161]​ | Acute Total Hip Arthroplasty for the Treatment of Acetabular Fractures: A Retrospective Study With a Six-Year Follow-Up | OS without control group | Greece | 14 |
| Sarlak et al. (2014) ​[160]​ | Management of acetabular fractures with modified posterior approach to spare external hip rotators | OS without control group | Turkey | 37 |
| Saterbak et al. (2000) ​[159]​ | Clinical Failure After Posterior Wall Acetabular Fractures: The Influence of Initial Fracture Patterns | OS with control group | USA | 42 |
| Schellmann et al. (1975) ​[158]​ | Ergebnisse nach operativer Wiederherstellung der Hüftpfanne [Results after surgical reconstruction of the acetabulum] | OS without control group | Germany | 26 |
| Schwab et al. (2011) ​[157]​ | The Use of Cervical Vertebrae Plates for Cortical Substitution in Posterior Wall Acetabular Fractures | OS without control group | USA | 23 |
| Selvaratnam et al. (2021) ​[167]​ | Outcomes of acute fix and replace in complex hip posterior fracture dislocations with acetabular fractures: a minimum of 3 years follow-up | OS without control group | UK | 14 |
| Senegas et al. (1980) ​[166]​ | Complex Acetabular Fractures: A Transtrochanteric Lateral Surgical Approach | OS without control group | France | 28 |
| Sharma et al. (2019) ​[165]​ | Shortterm followup of anterior and posterior both column fractures of acetabulum managed through both column plating | OS without control group | India | 10 |
| Sharma et al. (2023) ​[164]​ | Tricortical iliac crest graft as a salvageable option in the reconstruction of comminuted posterior wall acetabular fractures: our experience from a level 1 trauma centre | OS without control group | India | 14 |
| Shazar et al. (2014) ​[170]​ | Comparison of Acetabular Fracture Reduction Quality by the Ilioinguinal or the Anterior Intrapelvic (Modified Rives–Stoppa) Surgical Approaches | OS with control group | Israel | 225 |
| Shi et al. (2014) ​[171]​ | Radiographic analysis of the restoration of hip joint center following open reduction and internal fixation of acetabular fractures: a retrospective cohort study | OS without control group | China | 127 |
| Siebenrock et al. (2002) ​[169]​ | Surgical Dislocation of the Femoral Head for Joint Debridement and Accurate Reduction of Fractures of the Acetabulum | OS without control group | Switzerland | 12 |
| Siebenrock et al. (2006) ​[172]​ | Trochanteric Flip Osteotomy for Cranial Extension an Muscle Protection in Acetabular Fracture Fixation Using a Kocher-Langenbeck Approach | OS without control group | Switzerland | 10 |
| Singh et al. (2020) ​[183]​ | Clinico-Radiological Evaluation of Modified Stoppa Approach in Treatment of Acetabulum Fractures | OS without control group | India | 30 |
| Singh et al. (2022) ​[168]​ | THA in patients with neglected acetabular fractures | OS without control group | India | 49 |
| Soni et al. (2020) ​[182]​ | Combined Anterior Pelvic (CAP) approach for fracture acetabulum fixation - Functional outcome evaluation and predictors of outcome | OS without control group | India | 47 |
| Splavski et al. (2013) ​[181]​ | Reducing Pain and Improving Quality of Life for Patients Suffering the Acetabular Fracture | OS with control group | Croatia | 21 |
| Swartman et al. (2020) ​[180]​ | Minimally invasive surgical treatment of minimally displaced acetabular fractures does not improve pain, mobility or quality of life compared to conservative treatment: a matched-pair analysis of 50 patients | OS with control group | Germany | 25 |
| Swartman et al. (2021) ​[179]​ | Fracture reduction and screw position after 3Dnavigated and conventional fluoroscopyassisted percutaneous management of acetabular fractures: a retrospective comparative study | OS with control group | Germany | 37 |
| Tan et al. (2003) ​[176]​ | Open Reduction and Internal Fixation of Fractures of the Acetabulum – Local Experience | OS without control group | Singapore | 15 |
| Tang;  NCT01437150 (ongoing) ​[216]​ | Treatment of Acetabular Posterior Wall Fracture With Anatomical Locking Plate | RCT | China | 25 |
| Tidermark et al. (2003) ​[175]​ | Primary Total Hip Arthroplasty with a Burch-Schneider Antiprotrusion Cage and Autologous Bone Grafting for Acetabular Fractures in Elderly Patients | OS without control group | Sweden | 10 |
| Uchida et al. (2013) ​[177]​ | Fracture of the acetabulum: a retrospective review of ninety-one patients treated at a single institution | OS with control group | Japan | 71 |
| Ulrich et al. (1986) ​[178]​ | Primäre Alloarthroplastik bei Acetabulumfrakturen [Primary allo-arthroplasty in acetabulum fractures] | OS without control group | Germany | 15 |
| Ur Razaq et al. (2016) ​[174]​ | Outcome Of Two Column Acetabular Fractures Treated Operatively Through Single Posterior Approach | OS without control group | Pakistan | 25 |
| Verbeek et al. (2018) ​[173]​ | Long-term patient reported outcomes following acetabular fracture fixation | OS with control group | USA | 106 |
| Wan et al. (2022) ​[196]​ | The novel infra-pectineal buttress plates used for internal fixation of elderly quadrilateral surface involved acetabular fractures | OS without control group | China | 23 |
| Wang et al. (2016) ​[197]​ | Modified ilioinguinal approach in combined surgical exposures for displaced acetabular fractures involving two columns | OS with control group | China | 73 |
| Wang et al. (2023) ​[195]​ | Treatment of acetabular fracture involving anterior and posterior columns using a single pararectus approach: surgical experience and preliminary results | OS without control group | China | 58 |
| Weaver et al. (2018) ​[194]​ | Does Total Hip Arthroplasty Reduce the Risk of Secondary Surgery Following the Treatment of Displaced Acetabular Fractures in the Elderly Compared to Open Reduction Internal Fixation? A Pilot Study | OS with control group | USA | 70 |
| Wenzel et al. (2020) ​[193]​ | The Pararectus Approach in Acetabular Surgery: Radiological and Clinical Outcome | OS with control group | Germany | 61 |
| Wollmerstädt et al. (2020) ​[192]​ | Mortality, complications and long-term functional outcome in elderly patients with fragility fractures of the acetabulum | OS with control group | Germany | 109 |
| Wu et al. (2016) ​[190]​ | Comparison of open reduction and internal fixation in treatment of delayed and early acetabular fractures | OS with control group | China | 90 |
| Wu et al. (2020) ​[191]​ | Single Ilioinguinal Approach to Treat Complex Acetabular Fractures with Quadrilateral Plate Involvement: Outcomes Using a Novel Dynamic Anterior Plate–Screw System | OS without control group | China | 32 |
| Wu et al. (2020) ​[189]​ | Personalized Three-Dimensional Printed Anterior Titanium Plate to Treat Double-Column Acetabular Fractures: A Retrospective Case-Control Study | OS with control group | China | 43 |
| Xiao et al. (2021) ​[188]​ | Traditional versus mirror three-dimensional printing technology for isolated acetabular fractures: a retrospective study with a median follow-up of 25 months | OS with control group | China | 114 |
| Xu et al. (2023) ​[187]​ | Posterior wall fractures of the acetabulum: treatment using an anatomical plate through direct posterior approach | OS with control group | China | 41 |
| Yang et al. (2020) ​[185]​ | The Stoppa combined with iliac fossa approach for the treatment of both-column acetabular fractures | OS with control group | China | 76 |
| Yao et al. (2021) ​[186]​ | Internal fixation of anterior acetabular fractures with a limited pararectus approach and the anatomical plates:  preliminary results | OS without control group | China | 17 |
| Yavuz et al. (2022) ​[184]​ | The Impact of Surgical Approaches for Isolated Acetabulum Fracture on Sexual Functions: A Prospective Study | OS with control group | Turkey | 65 |
| Yu et al. (2004) ​[203]​ | Surgical treatment of displaced fractures of posterior column and posterior wall of the acetabulum | OS without control group | China | 11 |
| Yu et al. (2020) ​[204]​ | Traditional three-dimensional printing technology versus three-dimensional printing mirror model technology in the treatment of isolated acetabular fractures: a retrospective analysis | OS with control group | China | 146 |
| Zha et al. (2020) ​[205]​ | A new internal fixation technique for acetabular fractures involving the quadrilateral plate | OS without control group | China | 24 |
| Zhang, & Hou;  NCT03026868 (ongoing) ​[217]​ | Randomized Trails of Different Fixations for Acetabular Fracture Involving Quadrilateral Surface | OS without control group | China | *Target: 80* |
| Zhang et al. (2016) ​[202]​ | An Effective and Feasible Method, (Hammering Technique,) for Percutaneous Fixation of Anterior Column Acetabular Fracture | OS without control group | China | 16 |
| Zhang et al. (2019) ​[206]​ | Fixation of Displaced Acetabular Fractures With an Anatomic Quadrilateral Surface Plate Through the Stoppa Approach | OS without control group | China | 26 |
| Zheng et al. (2022) ​[201]​ | Application of computerized virtual preoperative planning procedures in comminuted posterior wall acetabular fractures surgery | OS with control group | China | 45 |
| Zinghi et al. (2003) ​[200]​ | Le fratture dell´acetabolo [Acetabluar fractures] | OS without control group | Italy | 477 |
| Zou et al. (2020) ​[199]​ | Therapeutic Effect of Acetabular Fractures Using the Pararectus Approach Combined with 3D Printing Technique | OS without control group | China | 33 |
| Zou et al. (2021) ​[198]​ | Clinical Results of Acetabular Fracture via the Pararectus versus Ilioinguinal Approach | OS with control group | China | 60 |

*Legend: RCT = Randomized controlled trial; OS = Observational study; USA = United States of America; UK = United Kingdom*
