# Supplementary material for: Developing a core outcome set for acetabular fractures: a systematic review (part I)
Source: Syst Rev. 2025 Apr 9;14:83. doi: 10.1186/s13643-025-02824-0 (PMC11983908; doi:10.1186/s13643-025-02824-0)
Supplement: Supplementary file 5 — Additional file 5. Outcome categorization. [file 13643_2025_2824_MOESM5_ESM.docx]

**Outcome categorization**

| **Core area** | **Outcome domain** | **Number of studies (%)** | **Number of unique outcomes** | **Unique outcomes** |
| --- | --- | --- | --- | --- |
| **Death** | Mortality/ survival | 21 (10.88) | 1 | mortality |
| **Physiological/ clinical** | Cardiac | 5 (2.59) | 1 | cardiovascular complications |
|  | Gastrointestinal | 14 (7.25) | 6 | damage to the sphincter, gastrointestinal bleeding, hernia, ileus, peritoneal injury, visceral injury |
|  | General | 162 (83.94) | 16 | cachexia, clinical and radiographic examination, collapse, complex regional pain syndrome, fluid overload, general health, hypovolemic shock, independent in continence, ischemic necrosis of abdominal or pelvic organs, pain, perioperative physiological variables, qualitative outcome, recreation, sleep/rest, use of medication for pain, vitality |
|  | Immune system | 1 (0.52) | 1 | inflammatory response |
|  | Infection and infestation | 105 (54.40) | 7 | deep infection, hematogenous infection with Methicillin in Resistant Staphylococcus Aureus, infected hematoma with coagulase-negative Staphylococcus aureus, infection, intra-articular infection, sepsis, superficial infection |
|  | Injury and poisoning | 9 (4.66) | 3 | damage to the peri-pelvic organs, iatrogenic neurovascular injury, spinal cord ischemic injury |
|  | Musculoskeletal and connective tissue | 186 (96.37) | 78 | accuracy of screw insertion, acetabular component inclination, acetabular component migrations, acetabular or weight-bearing dome, appearance the hip joint, arthritis, associated focal articular lesions of the femoral head, associated upper or lower limb fracture, atrophy, atrophy of the rectus, avascular necrosis of chondrolysis, avascular necrosis of the femoral head, bone union, bone union time, broken guidewire of the cannulated screw, changes in the angular position, component migration, contracture of the knee, deformity, degenerative changes, delayed union, dislocation, epiphyseal necrosis, femoral component migration, femoral fracture, fixation failure, groin discomfort in the area of anterior plating, hematoma, heterotopic ossification, hip arthrofibrosis, implant failure, implant loosening, intra-articular fragment, intra-articular screw penetration, intra-articular hardware, joint space narrowing, kinetic variables, limb length, location of any osteolytic lesions, loss of reduction, lumbar lordosis, malposition of the implant, marginal impaction, medialization, nonunion, osteolysis of the acetabulum, osteomyelitis, periosteosynthetic fracture, periprosthetic fracture, posterior re-subluxation of the hip, prosthetic joint infection, protrusion, quality of reduction, quality of reduction of trochanter, radiological outcome, range of motion, rapid erosion of the femoral head, rectus abdominis muscle paralysis, removal of fixation devices, residual instability, residual gap, rim distance, screw irritation, screw migration, screw position, secondary displacement, soft tissue complication, spatiotemporal variables, stability of the femoral and acetabular components, stiffness, subsidence of the femoral component, distance between subchrondal bone and the most inner screw, screw numbers on major fragment, trendelenburg sign, trochanter migration, trochanteric bursitis, wear on polyethylene insert, weight-bearing |
|  | Nervous system | 100 (51.81) | 25 | dysesthesia in femoral cutaneous nerve territory, femoral nerve injury, femoral nerve palsy, foot drop, iatrogenic nerve injury, iatrogenic nerve palsy, iatrogenic sciatic nerve injury, iatrogenic sciatic nerve symptom, iatrogenic sciatic nerve palsy, lateral femoral cutaneous nerve injury, lateral femoral cutaneous nerve palsy, nerve injury, nerve palsy, neurological complication, neurological status, obturator nerve injury, obturator nerve palsy, peroneal nerve injury, peroneal nerve palsy, sciatic nerve injury, sciatic nerve neuropraxia, sciatic nerve palsy, sensory function, stroke, symptoms of nerve stimulation |
|  | Renal and urinary | 16 (8.29) | 5 | kidney injury, renal impairment, urinary bladder rent, urinary tract infection, urosepsis |
|  | Psychiatric | 34 (17.62) | 2 | delirium, mental status |
|  | Respiratory, thoracic and mediastinal | 14 (7.25) | 3 | pneumonia, pulmonary infarction, pulmonary infection |
|  | Skin and subcutaneous tissue | 27 (13.99) | 13 | decubitus, delayed wound healing, fat liquefaction, graft donor site complications, incision length, Morel–Lavallee lesion, seroma, skin problem, wound cellulitis, wound complications, wound dehiscence, wound healing, wounds healing time |
|  | Vascular | 96 (49.74) | 23 | arterial embolism, arterial puncture, atrogenic nick in the femoral vein, bleeding from the left hypogastric vein, blood loss, corona mortis injury, deep vein thrombosis, femoral artery perforation, femoral vascular injury, iatrogenic obturator artery injury, iatrogenic vascular injury, iliac artery embolism, iliac vein injury, iliac vein thrombosis, obturator artery injury, pulmonary embolism, superior gluteal artery injury, thromboembolic event, thrombophlebitis, thrombosis, transient ischemic attack, vascular injury, vascularity of the femoral head |
| **Life impact** | Physical functioning | 161 (83.42) | 30 | ability to access transportation, ability to climb stairs, ability to do housework, ability to go to the toilet, ability to shop for household, ability to sit, ability to stand up, ability to transfer, ability to walk, ability to walk independently, ability to wash or dress, able to resume driving a 2 wheeler, able to resume driving a 4 wheeler, activities of daily living, activity, balance, cross-legged sitting, erectile dysfunction, function, function of arm and hand, hand and wrist function, impact on sexual function, independent in feeding, lifting object, limping, mobility, running, self-care, time to full weight-bearing, time to return to activities of daily life |
|  | Social functioning | 18 (9.33) | 2 | communication, social functioning |
|  | Role functioning | 24 (12.44) | 4 | ability to work, maintenance in role model (emotional or physical), return to work, time to return to work |
|  | Emotional functioning/ wellbeing | 2 (1.04) | 5 | arousal, orgasm, psychological decompensation, satisfaction, sexual desire |
|  | Cognitive functioning | 1 (0.52) | 1 | cognitive dysfunction |
|  | Delivery of care | 1 (0.52) | 1 | satisfaction with medical care |
| **Resource use** | Economic | 45 (23.32) | 16 | 3D printing time, arthroplasty time, fixed with reconstruction plates, fixed with screw only, fluoroscopy time, follow-up time, instrumentation time, learning curve, mean time for performing temporary balloon occlusion of the abdominal aorta, numbers of attempts of guide wire, number of fluoroscopic screenings, number of the approach, operation time, preoperative software time, surgical details, time for patient-specific plate preparation |
|  | Hospital | 16 (8.29) | 5 | hospital readmission, intensive care unit stay in days, intensive care unit treatment, length of hospital stay, time to surgery |
|  | Need for further intervention | 62 (32.12) | 12 | blood transfusion, conversion to total hip arthroplasty, fixation of periprosthetic fracture, hematoma requiring revision, hemorrhages requiring transfusion, intra operative bleeding requiring surgical intervention, need for embolization of injured blood vessel, presence of loose bodies inside the joint space requiring surgical removal, reoperation, revision, secondary arthroscopy, time to total hip arthroplasty |
|  | Societal/ carer burden | 4 (2.07) | 2 | discharge location, return to their home |
| **Adverse events** | Adverse events/ effects | 17 (8.81) | 4 | complications, complications associated with arthroscopy, non-surgical complications, surgical complication |
