# Supplementary material for: Developing a core outcome set for acetabular fractures: a systematic review (part I)
Source: Syst Rev. 2025 Apr 9;14:83. doi: 10.1186/s13643-025-02824-0 (PMC11983908; doi:10.1186/s13643-025-02824-0)
Supplement: Supplementary file 6 — Additional file 6. Measurement instruments. [file 13643_2025_2824_MOESM6_ESM.docx]

**Measurement instruments**

| Measurement instrument (including modifications) | Number of studies (%) | Study references |
| --- | --- | --- |
| Activity of Daily Living | 2 (1.28) | [175, 216] |
| EuroQol-5D | 12 (7.69) | [23, 53, 59, 68, 70, 115, 146, 156, 161, 175, 209, 214] |
| Functional Independence Measure (FIM) | 1 (0.64) | [216] |
| Female Sexual Function Index | 1 (0.64) | [184] |
| Harris Hip Score | 57 (36.54) | [23, 27, 32, 36, 37, 43, 44, 47, 49, 51–54, 57, 60, 61, 69, 71, 72, 77, 78, 80, 81, 83, 85–87, 89, 94, 100, 107, 110, 114, 117, 119, 125, 133, 137–139, 141, 148, 155, 161, 162, 165, 168, 174, 175, 180, 188, 194, 197, 202–204, 207, 208, 211] |
| Merle d'Aubigné  Method | 91 (58.33) | [24–26, 29, 31, 34, 36, 39, 41, 42, 45, 47, 48, 51, 57, 58, 62, 63, 65, 66, 69, 74–76, 79, 81, 83–85, 88, 91, 95, 96, 101, 102, 105, 106, 108, 112, 113, 115, 118, 121, 123, 124, 126, 127, 130, 131, 134–137, 141, 142, 144, 147, 149, 150, 152, 154, 159, 160, 164, 166, 169, 172, 176–178, 180, 182–187, 189–192, 195, 196, 198–201, 205, 206, 208, 212, 215] |
| Musculoskeletal Function Assessment Instrument (MFA) | 2 (1.28) | [90, 159] |
| Oxford Hip Score | 11 (7.05) | [70, 92, 97, 99, 104, 115, 132, 143, 161, 209, 214] |
| 8-Item Short Form Survey (SF-8) | 2 (1.28) | [140, 146] |
| 12-Item Short Form Survey (SF-12) | 6 (3.85) | [83, 94, 117, 119, 173, 180, 208] |
| 36-Item Short Form Survey (SF-36) | 10 (6.41) | [42, 52, 61, 90, 100, 102, 122, 137, 194, 216] |
| Short Musculoskeletal Function Assessment (SMFA) | 5 (3.21) | [38, 53, 60, 102, 173, 207] |
| Tinetti Balance Test | 1 (0.64) | [156] |
| Workplace Activity Limitation Survey (WALS) | 1 (0.64) | [119] |
| Western Ontario and McMaster Universities Arthritis Index (WOMAC) | 3 (1.92) | [34, 140] |
| Disability Rating Index | 1 (0.64) | [209] |
| Patient Satisfaction (Pain & ability to walk) (Mitsionis_2012) | 1 (0.64) | [123] |
